# Global Shifts in Gene Expression Profiles Accompanied with Environmental Changes in Cnidarian-Dinoflagellate Endosymbiosis

**DOI:** 10.1534/g3.118.201012

**Published:** 2019-05-16

**Authors:** Yuu Ishii, Shinichiro Maruyama, Hiroki Takahashi, Yusuke Aihara, Takeshi Yamaguchi, Katsushi Yamaguchi, Shuji Shigenobu, Masakado Kawata, Naoto Ueno, Jun Minagawa

**Affiliations:** * Graduate School of Life Sciences, Tohoku University, Sendai, Miyagi, Japan; † Division of Morphogenesis, National Institute for Basic Biology, Okazaki, Aichi, Japan; ‡ Department of Basic Biology, School of Life Science, SOKENDAI (The Graduate University for Advanced Studies), Okazaki, Aichi, Japan; § Division of Environmental Photobiology, National Institute for Basic Biology, Okazaki, Aichi, Japan; ** Functional Genomics Facility, National Institute for Basic Biology, Okazaki, Aichi, Japan

**Keywords:** Symbiosis, Symbiodiniaceae, Cnidarians, RNAseq, Lysosome

## Abstract

Stable endosymbiotic relationships between cnidarian animals and dinoflagellate algae are vital for sustaining coral reef ecosystems. Recent studies have shown that elevated seawater temperatures can cause the collapse of their endosymbiosis, known as ‘bleaching’, and result in mass mortality. However, the molecular interplay between temperature responses and symbiotic states still remains unclear. To identify candidate genes relevant to the symbiotic stability, we performed transcriptomic analyses under multiple conditions using the symbiotic and apo-symbiotic (symbiont free) *Exaiptasia diaphana*, an emerging model sea anemone. Gene expression patterns showed that large parts of differentially expressed genes in response to heat stress were specific to the symbiotic state, suggesting that the host sea anemone could react to environmental changes in a symbiotic state-dependent manner. Comparative analysis of expression profiles under multiple conditions highlighted candidate genes potentially important in the symbiotic state transition under heat-induced bleaching. Many of these genes were functionally associated with carbohydrate and protein metabolisms in lysosomes. Symbiont algal genes differentially expressed *in hospite* encode proteins related to heat shock response, calcium signaling, organellar protein transport, and sugar metabolism. Our data suggest that heat stress alters gene expression in both the hosts and symbionts. In particular, heat stress may affect the lysosome-mediated degradation and transportation of substrates such as carbohydrates through the symbiosome (phagosome-derived organelle harboring symbiont) membrane, which potentially might attenuate the stability of symbiosis and lead to bleaching-associated symbiotic state transition.

Coral reefs provide habitats for diverse marine animals, especially in oligotrophic tropical and subtropical oceans. These ecosystems rely on the stable endosymbiosis (intracellular symbiosis) between the dinoflagellate algae in the family Symbiodiniaceae and their host cnidarian animals (*e.g.*, coral, sea anemone, and jellyfish). The symbiosis between animal hosts and Symbiodiniaceae algae likely evolved multiple times independently in different lineages (Mies *et al.* 2017). It has been proposed that recent global climate change can cause elevated water temperature and consequently the collapse of the symbiosis, known as ‘bleaching’ (Hughes *et al.* 2017).

Bleaching is a consequence of physiological responses to environmental changes. A major form of bleaching is induced by elevated temperature, or heat stimulus, called heat-induced bleaching (HIB); however, bleaching can also be induced by other factors (*e.g.*, aberrant light condition, salinity, and nutrients) (Weis 2008). Two kinds of mechanisms have been proposed to be involved in the bleaching processes. One mechanism is the loss of pigments by symbionts, which causes the apparent whitening of the cnidarian host’s body color but does not necessarily affect the number of symbiont cells within the host (Takahashi *et al.* 2008). The other type of bleaching is the loss of algae by the host, which can not only change the coloration but, more importantly, affect the physiological conditions of the host cells (Weis 2008).

In the cnidarian-algal relationship, symbionts are maintained in a host-derived phagosomal compartment within gastrodermal cells of the host, called symbiosome; symbiosomes have a low internal pH and are acidified by proton ATPases, as in other host cell compartments (*e.g.*, lysosomes, endosomes, and vacuoles) (Wakefiel and Kempf 2001; Barott *et al.* 2015). Multiple routes to ‘loss of algae’ have been proposed, for example symbiont cell degradation within the symbiosome via fusion with lysosome and/or autophagosome, exocytosis from the host cell, and host cell death and detachment from the tissue (Weis 2008; Bieri *et al.* 2016). Although it is critical to understand how the symbiosomes are regulated under normal and stress conditions, host-symbiont communication through the symbiosome membrane at the molecular and genetic levels remains uncharacterized (Davy *et al.* 2012; Bieri *et al.* 2016).

Recently, a number of whole genome-level analyses (*e.g.*, genomics, transcriptomics, and metabolomics) have been employed to clarify the molecular mechanisms of the symbiosis between cnidarian hosts and Symbiodiniaceae symbionts by using corals and sea anemones. Among these, the sea anemone *Exaiptasia diaphana* (formerly *Aiptasia* sp.) (Daly and Fautin 2018) is an emerging model cnidarian animal; it is experimentally feasible to induce symbiotic and apo-symbiotic (*i.e.*, symbiont free) states reversibly by inoculation with free-living Symbiodiniaceae algae and removal of the symbionts under aberrant temperature conditions in the laboratory (Sunagawa *et al.* 2009; Lehnert *et al.* 2012; Grajales and Rodríguez 2014; Baumgarten *et al.* 2015; Weis *et al.* 2008).

Previous transcriptomic and proteomic studies reported that in host sea anemones many metabolic pathways and cellular processes were affected by symbiosis based on the comparison between the symbiotic and apo-symbiotic states (Kuo *et al.* 2004; Rodriguez-Lanetty *et al.* 2006; Ganot *et al.* 2011; Lehnert *et al.* 2014; Oakley *et al.* 2016; Matthews *et al.* 2017). Meanwhile, a proteomic analysis using *E. diaphana* has identified a number of metabolic pathways located in cellular compartments (*e.g.*, endoplasmic reticulum) as essential in the cellular response to heat stress (Oakley *et al.* 2017). Nevertheless, the interplay between symbiotic states and heat stress responses has not been well studied at the molecular level via genome-wide analyses.

Here, we have conducted transcriptomic analyses under different temperature conditions using *E*. *diaphana* in its different symbiotic states, allowing us to (1) characterize the differences in the host gene expression profiles between symbiotic and apo-symbiotic individuals in response to heat stress, (2) identify host genes and their functions, which are potentially associated with the process of heat-induced bleaching, and (3) identify symbiont gene expression changes induced by heat stress *in hospite* (in the host body).

## Material and Methods

### Strains and culture conditions

All anemones used for this study were from a clonal sea anemone *E. diaphana* strain H2 harboring a homogenous population of *Breviolum* (formerly *Symbiodinium* clade B) symbionts (Xiang *et al.* 2013). Symbiotic and apo-symbiotic *E. diaphana*, generous gifts from Profs. John R. Pringle and Arthur R. Grossman, were maintained at a density of 20 to 50 animals per plastic cage (12 × 22 × 13 cm, length × width × height) and fed with *Artemia* sp. (A&A Marine, Utah, USA) every three or four days. The symbiotic cultures were grown in circulating artificial sea water (ASW) at 25° with fluorescent light irradiation at 60 µmol s^-1^ m^-2^ with 12 h light:12 h dark cycle. The apo-symbiotic cultures were grown in circulating ASW at 25° without fluorescent light irradiation.

### Experimental design

Incubating conditions were designed to detect changes in an early phase of heat stress responses, based on previous studies that suggested 24 h incubation at elevated temperature induced a change in the photosynthetic activity but not the number of symbiont cells in the symbiosis between *E. diaphana* and Symbiodiniaceae symbionts (Hillyer *et al.* 2016; Oakley *et al.* 2017). Prior to heat stress experiments, apo-symbiotic individuals were also incubated under the light-dark cycle for one week, and used for experiments only when they showed no sign of possible repopulation by symbiotic algae, *i.e.*, neither algal cell bodies nor particles displaying chlorophyll fluorescence was observed under bright-field and fluorescence microscopes. During the experimental trial, symbiotic and apo-symbiotic *E. diaphana* individuals were cultured separately at two temperatures (25° or 33°) for 24 h (6 h light: 12 h dark: 6 h light) at a density of 4 to 5 animals per round plastic case (4 × 9 cm, height × diameter), filled with ASW, and three individuals per treatment were sampled from a plastic case for RNAseq. To minimize contamination from *Artemia* RNA, anemones were not fed for 1 week prior to sampling.

### Protein quantification and cell count

To measure the number of symbiont cells per host protein, each symbiotic *E. diaphana* was put into 50 µl PBS in a microtube and ground with a Biomasher II pestle (Nippi, Japan) until debris was no longer observed. Symbiont cell densities in the host cell suspension were quantified using improved Neubauer hemocytometer (Fukaekasei, Japan), with a minimum of four replicate cell counts per sample. Cell density was normalized to soluble protein content, which was assessed by using TAKARA BCA Protein Assay kit (Takara Bio, Japan) with the supernatant centrifuged (16,000 × *g* for 1 min) host fractions (quintuplicate measurements). Mean estimates with standard error (SEM) were calculated based on single measurements using five individuals per temperature treatment, which were separate from the samples used for transcriptome analysis.

### Photosynthesis and respiration activity assay

Maximum quantum yield of photosystem II (Fv/Fm) was measured with a diving pulse amplitude modulated (PAM2500) fluorometer (Walz, Effeltrich, Germany), following a 30 min dark adaptation. PAM settings were adjusted with Ft ≤ 0.3. Photosynthesis and respiration rates were measured with a Clark-type oxygen electrode (Hansatech Instruments, Norfolk, UK) in a closed cuvette in the light at 1,000 µmol m^-2^ s^-1^ photons at 25°. Individuals were preincubated in the dark for 10 min and then exposed to saturating light for 20 min. The respiration rate was calculated from the dark-phase oxygen consumption rate. The photosynthesis rate was calculated by subtracting the respiration rate from the light-phase oxygen evolution rate. Mean estimates with SEM were calculated based on single measurements using four individuals per temperature treatment, separate from the transcriptome analysis.

### RNA extraction and sequencing

Three symbiotic or apo-symbiotic individuals per temperature condition were put into RNAlater RNA Stabilization Solution (Thermo Fisher Scientific, Massachusetts, USA) in a microtube (one individual per tube) and stored at 4°, then the solution was replaced with 480 µl of Trizol reagent (Thermo Fisher Scientific, Massachusetts, USA). The samples were ground with a motor-assisted pestle, Biomasher II (Nippi, Japan) until debris was no longer observed. RNA extraction with Trizol reagent was conducted according to the manufacturer’s instruction. The quality and quantity of RNA were verified using Agilent RNA 6000 Nano Kit on Agilent Bioanalyzer (Agilent Technologies, California, USA) and Nanodrop spectrophotometer (Thermo Fisher Scientific, Massachusetts, USA), respectively. One µg of total RNA from each individual was subjected to library preparation with no size selection using TruSeq RNA Sample Prep v2 Kits (Illumina, California, USA) according to the manufacturer’s protocol (#15026495 Rev. D). These mRNA libraries were sequenced on Illumina Hiseq1500 with 100-mer paired-end sequence.

### Transcriptome analysis

Reads from a total of 12 libraries each were obtained, trimmed, and filtered by trimmomatic option of Trinity program (Grabherr *et al.* 2011); ‘PwU’ output reads were used for analysis. Sequence reads were mapped against genome assemblies of *E. diaphana* (*Aiptasia* sp.) (Baumgarten *et al.* 2015) and *Breviolum* (formerly *Symbiodinium*) *minutum* (Shoguchi *et al.* 2013) using TopHat2 with default setting (Kim *et al.* 2013). Mapped transcripts, or fragments per kilobase of exon per million mapped fragments (FPKM) values (Mortazavi *et al.* 2008), were collected using Cufflinks Ver. 2.2.1 (Trapnell *et al.* 2010) and converted to read counts per gene using HTSeq (Anders *et al.* 2015). To obtain a visual overview of the effect of temperature treatments and host symbiotic states (symbiotic or apo-symbiotic) on global gene expression patterns, principal component analysis (PCA) was performed on the calculated FPKM values using the function “prcomp” in R (http://www/r-project.org/). Genes expressed in at least one individual were included in the PCA analysis. The count data were normalized with the R package “TCC” (Sun *et al.* 2013) and differential gene expression analysis was conducted with the “edgeR” (Robinson *et al.* 2010) analysis, implemented within the TCC package. To define differentially expressed genes (DEGs), or genes with statistically significant differences in expression between the two temperatures treatments or two host symbiotic states, the false discovery rate (FDR), or q-value, of 0.001 was used as cutoff.

Gene ontology (GO) term enrichment analysis was performed using “GOseq” package in R (Young *et al.* 2010). To annotate each *E. diaphana* gene with GO terms, BLASTp search was performed (E value cutoff, 10^−4^) against the *Ciona intestinalis* protein dataset using all of the *E. diaphana* protein dataset as query, resulting in 15279 orthologs. For symbiont genes, BLASTp search (E value cutoff, 10^−4^) against *Arabidopsis thaliana* using all of the *B. minutum* protein dataset as query, resulting in 15407 orthologs. Among many reference genomes available from related taxa, advantages to use the *C. intestinalis* and *A. thaliana* genomes are: (1) These species have long histories of *in vivo* gene function analyses and more empirical GO annotation data, (2) *C. intestinalis* is a relatively closely related lineage to cnidarians and useful in similarity-based homolog searches, (3) The *A. thaliana* genome is one of the most useful and well-documented among photosynthetic species to analyze photosynthesis-related functions. Overrepresented p-values produced by GOseq were adjusted using the Benjamini-Hochberg correction (Benjamini and Hochberg 1995). The adjusted p-value (q-value) of 0.05 was used to define enriched GO terms. Presence–absence matrix of genes associated with enriched GO terms, with dendrogram showing heatmap clustering and a table showing log fold-change (logFC) values output by TCC, was generated using “Heatmap3” package in R.

### Phylogenetic analysis and localization prediction

Filtered reads of 12 libraries were *de novo* assembled using Trinity program (Grabherr *et al.* 2011). A contig containing 28S large subunit ribosomal RNA gene (LSU rDNA) sequence was searched by BLASTn and aligned with other sequences using the Symbiodiniaceae LSU rDNA data in a previous study (LaJeunesse *et al.* 2018). The manually curated alignment was used to reconstruct phylogenetic trees using IQ-TREE with the TIM3+F+I+G4 model which ModelFinder selected as the best model by likelihood comparison based on the Bayesian information criterion (Nguyen *et al.* 2015; Kalyaanamoorthy *et al.* 2017). The *NPC2* gene sequences were translated into proteins and used for multiple sequence alignment and phylogenetic analysis as previously described (Maruyama *et al.* 2011), with the following modifications: homologous sequences automatically collected from the GenBank database were manually curated and selected for multiple alignments, and IQ-TREE was used to reconstruct phylogenetic trees using the LG+F+G4 model selected as described earlier. DHE tree was generated in the same way except using LG+I+G4. For predicting protein subcellular localization, iPSORT (Bannai *et al.* 2002) was used to predict a signal peptide or mitochondrial targeting peptide in a protein sequence, and MemPype (Pierleoni *et al.* 2011) was used to annotate eukaryotic membrane proteins.

### Data availability

The dataset supporting the results of this article is included within the article and its supplemental material files. The raw data from the symbiotic and apo-symbiotic *E. diaphana* RNAseq have been submitted to the DDBJ/EMBL-EBI/GenBank under the BioProject accession number PRJDB7145. Supplemental material available at FigShare: https://doi.org/10.25387/g3.7936622.

## Results

### Global gene expression patterns

RNAseq produced 507,857,846 reads from apo-symbiotic (‘Apo’) and symbiotic (‘Sym’) *E. diaphana* individuals with triplicates for each culture condition (*i.e.*, 3 Apo and 3 Sym samples under normal temperature [25°, called ‘Norm’] and elevated temperature [33°, called ‘Heat’] condition). The total number of mapped reads of the triplicates onto the dataset generated by combining all the scaffolds of the host and symbiont reference genomes were 14,573,222 reads for Sym-Norm, 13,304,783 for Sym-Heat, 20,692,119 for Apo-Norm, and 19,442,072 for Apo-Heat. Reads likely from co-cultured, or ’contaminating’, microbes included in the raw data were filtered out in this mapping process. Overall, we obtained the FPKM and count values of genes in the *E. diaphana* genomes under each of four conditions (*i.e.*, Apo-Norm, Apo-Heat, Sym-Norm, and Sym-Heat) and the *B. minutum* genome for two conditions (*i.e.*, Norm and Heat). In the symbiotic and apo-symbiotic samples, the proportions of reads mapped onto the symbiont genome sequences were about 10% and less than 1%, respectively.

**Figure 1:**
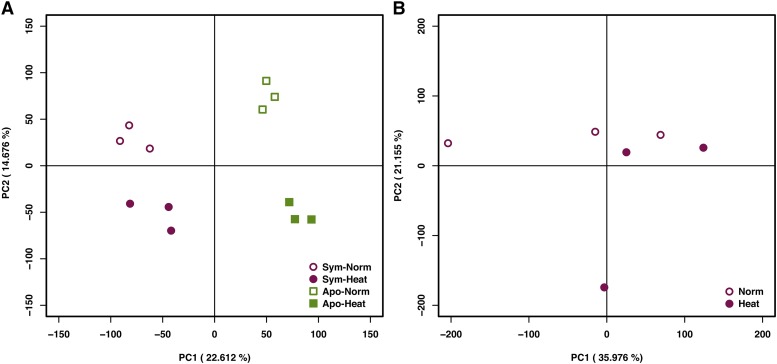
Global gene expression patterns in *E. diaphana* and the symbionts. A. PCA of the *E. diaphana* transcriptomes. B. PCA of the symbiont transcriptomes *in hospite*.

We conducted principal component analysis (PCA) of the gene expression patterns using the FPKM data mapped to the host *E. diaphana* or the *B. minutum* genome. In *E. diaphana*, the first principle component (PC1) represented the effect of symbiotic states (*i.e.*, apo-symbiotic or symbiotic), whereas PC2 represented those of temperature on gene expression levels (Figure 1A). The analysis separated the four conditions with no overlap (Figure 1A). In symbionts, the gene expression patterns in Norm and Heat thermal treatments were weakly separated along the PC2 axis (Figure 1B).

For the host sea anemone transcriptome analysis, the *E. diaphana* genome was used as a reference (Baumgarten *et al.* 2015). For the symbiont transcriptome, the *B. minutum* (formerly *S*. *minutum*, clade B) genome (Shoguchi *et al.* 2013) was used as a reference, as we found only one contig matching to 28S large subunit ribosomal RNA gene in our data, which formed a clade with *Breviolum* sequences (Figure S1).

The exposure to the elevated temperature for one day resulted in no apparent symptom of bleaching and no significant decline in the number of algal cells (Figure S2A), maximum quantum yield of photosystem II (Figure S2B), or photosynthesis rates (Figure S2C); however, respiration rates were decreased after the heat treatment (Figure S2D).

### E. diaphana DEGs

To detect genes differentially expressed between the test conditions in reference to previous studies, we used the FDR of 0.001 as the threshold, which is more stringent than the values used in other studies (*e.g.*, FDR of 0.05). For reference purpose, we compared the expression patterns of the Npc2-type sterol transporter gene family. To our knowledge, *NPC2* is one of the few examples of which the gene expression patterns were differentially regulated dependent on the symbiosis state in multiple cnidarian species in previous studies (Lehnert *et al.* 2014; Dani *et al.* 2014; Baumgarten *et al.* 2015; and references therein). In our data, out of six *NPC2* homologs, five genes were differentially expressed and all up-regulated in the Sym-Norm relative to the Apo-Norm condition (Figure S3, S4); thereby, results were consistent with previous reports (Lehnert *et al.* 2014). Furthermore, four genes among those five were significantly down-regulated in the Sym-Heat compared to the Sym-Norm group (Figure S3, S4).

**Figure 2:**
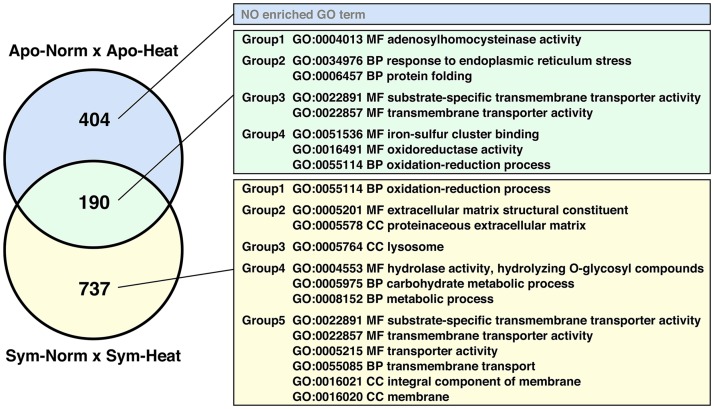
*E. diaphana* genes differentially expressed under heat stress in symbiotic and apo-symbiotic states. Venn diagram presents comparisons of the numbers of DEGs in each symbiotic state. Enriched GO terms also are shown for each compartment of the diagram. BP, biological process; CC, cellular component; MF, molecular function.

By comparing Apo-Norm and Apo-Heat test groups, we identified 594 genes as DEGs, whereas the Sym-Norm *vs.* Sym-Heat comparison resulted in 927 DEGs (Figure 2), which we call here two groups of heat-responsive DEGs (HR-DEGs). Between both groups, 190 genes were shared, called ‘shared HR-DEGs’. Gene expression regulation of the shared HR-DEGs were conserved between the symbiotic states, as the majority of HR-DEGs were down-regulated at an elevated temperature compared to the normal temperature in symbiotic and apo-symbiotic individuals (Figure S5). Heat shock proteins (HSPs) have been reported to be activated in some coral-symbiont systems under elevated temperature (DeSalvo *et al.* 2008; 2010). In *E. diaphana*, only *H90A1* was a shared HR-DEG, while *HS71A*, *HSP7C*, *HSP97*, and *HSP7C* were detected as unique HR-DEGs only found in the symbiotic individuals, and *CH10* and *AHSA1* only found in the apo-symbiotic state (Figure S6).

GO term enrichment analysis detected eight GO terms enriched in 190 shared HR-DEGs and 13 terms in 737 HR-DEGs unique to the symbiotic individuals (Figure 2, Table S1, Table S2). No GO term was enriched in 404 HR-DEGs unique to the apo-symbiotic individuals (Figure 2, Table S3). We analyzed these genes associated with the enriched GO terms by mapping them onto presence–absence matrices. The enriched GO terms of 190 shared HR-DEGs could be divided into four groups by heat map clustering for descriptive purposes (Figure S5). Groups 1, 2, 3 and 4 were related to methylation, protein folding in ER, transmembrane transport, and oxidation-reduction, respectively. The enriched GO terms of the HR-DEGs unique to the symbiotic individuals were partially overlapped with the ones for the shared HR-DEGs, *i.e.*, oxidation-reduction (Group 1), transmembrane transport (Group 5) (Figure S7).

**Figure 3:**
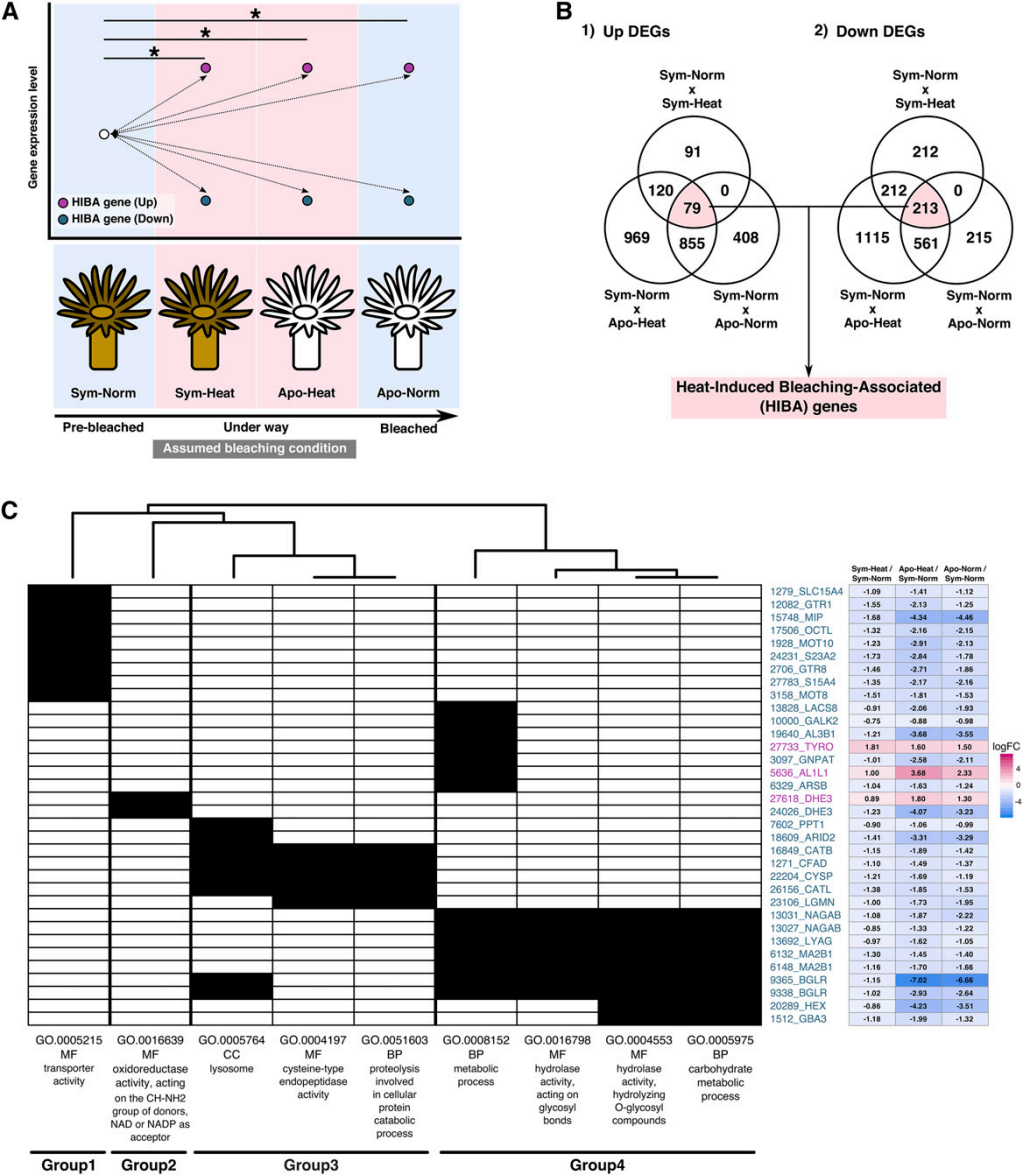
Expression patterns of HIBA genes. A. Conceptual representation of HIBA gene definition. HIBA genes are defined as DEGs of which all the expression levels in Sym-Heat, Apo-Heat, and Apo-Norm have been changed in the same direction in comparison with Sym-Norm. Assumed bleaching conditions are shown along with sample conditions. Asterisks indicate significantly differential gene expression. B. Venn diagram presents the numbers of DEGs in multiple comparisons. ‘Up’ and ‘Down’ DEGs indicate the ones up-regulated and down-regulated relative to Sym-Norm, respectively. C. Presence–absence matrix of HIBA genes associated with enriched GO terms. HIBA genes are shown with ‘AIPGENE’ gene IDs and putatively annotated gene names on the vertical axis, with a heat map showing gene expression levels as log FC values. Gene IDs shown in magenta and teal blue are up- and down-regulated genes relative to Sym-Norm. Enriched GO terms are shown with GO ID, GO category and description on the horizontal axis, with a clustering based on the genes presented in each GO term column. Closed and open cells indicate the presence and absence of the association with GO terms.

Considering HIB can have a major impact on coral bleaching in nature (Weis *et al.* 2008), we collected the DEGs that could potentially be associated with the HIB process. The four culture conditions introduced in this study can be assumed to mimic steps of a HIB process (Figure 3A): Sym-Norm (steady state prior to HIB), Sym-Heat (temperature elevation), Apo-Heat (heat-induced collapse of symbiosis), and Apo-Norm (steady state after HIB). In addition, the expressions of genes relevant to HIB can be considered to be altered irreversibly as the process progresses. We detected DEGs of which the expression levels were changed in the same direction (*i.e.*, either up- or down-regulated) in response to temperature elevation (Sym-Heat relative to Sym-Norm), symbiotic state transition (Apo-Norm relative to Sym-Norm), and a combination of those (Apo-Heat relative to Sym-Norm); these were called HIB-associated (HIBA) genes (Figure 3A). We identified 292 HIBA genes (Figure 3B, Table S4) and detected nine enriched GO terms associated with the HIBA genes, which were classified into four groups: transporter (Group 1), oxidation-reduction (Group 2), lysosome (Group 3), and carbohydrate metabolism (Group 4) (Figure 3C).

### Symbiont DEGs

**Figure 4:**
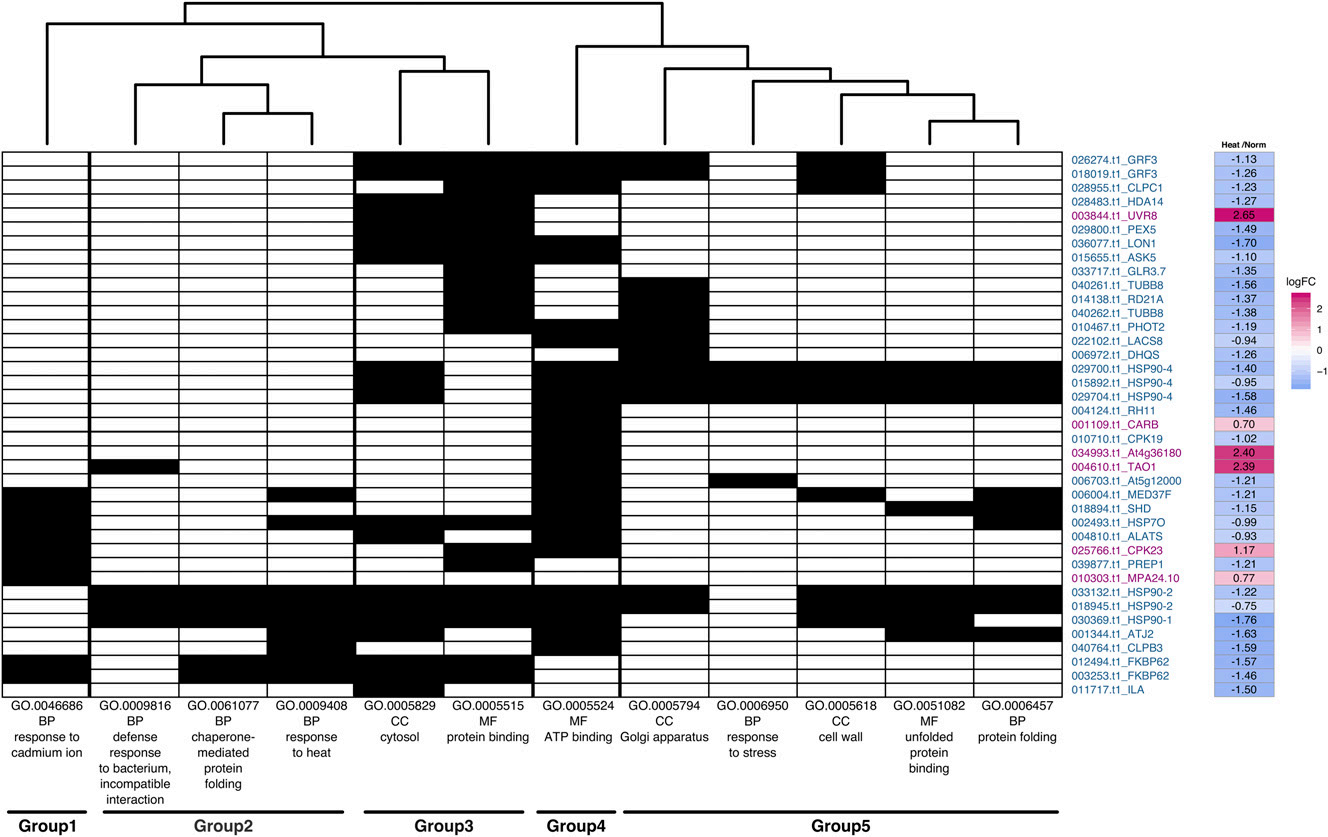
Presence–absence matrix and expression levels of HR-DEGs in symbionts. Symbiont HR-DEGs associated with enriched GO terms are shown as in Figure 3C, with the *B. minutum* gene IDs and putatively annotated gene names. Gene IDs shown in magenta and teal blue are up- and down-regulated genes relative to symbiont Norm, respectively.

By comparing the *Breviolum* spp. symbiont Norm and Heat samples, we identified 124 genes as HR-DEGs in the symbionts and conducted the GO term enrichment analysis of these genes based on the *A. thaliana* genome annotation data (Table S5), resulting in 12 terms detected. Although a number of groupings were recognized on the presence–absence matrix (Figure 4), many of the symbiont HR-DEGs were associated with multiple enriched GO terms and the classifications were not straightforward. Group 1 was associated with response to cadmium ion, while Group 2, 3, 4, and 5 were heat response, cytosol, ATP-binding, and stress response, respectively. A number of the symbiont HR-DEGs were not associated with any enriched GO terms, but potentially relevant to heat stress response in the symbionts (See Discussion) (Table S5).

## Discussion

### The host transcriptomic differences between symbiotic and apo-symbiotic states in response to heat stress

Our results show that heat stress responses, at least at the transcriptomic level, are different depending on the symbiotic states. Incubation under the elevated temperature conditions (33° for 24 h) led to no apparent indication of loss of algae (Figure S2). Considering previous studies showing that the number of symbiont cells decreased over time with incubation at elevated temperatures (Dunn *et al.* 2004; Hawkins *et al.* 2013), the Sym-Heat transcriptome in this study is most likely to reflect the very early phase of environmental response by the host *E. diaphana* prior to, rather than in process of, bleaching. The transcriptome profiling analyses revealed that both the symbiotic states and the thermal treatment had clear and substantially divergent effects on global gene expression patterns in the host (Figure 1A, 2), which is consistent with previous reports that symbiotic states are associated with different transcriptomic, proteomic, and metabolic profiles under normal growth conditions (Mitchelmore *et al.* 2003; Lehnert *et al.* 2014; Oakley *et al.* 2016). The *E. diaphana* individuals used for RNAseq were cultured by treatment and thus the conditions were not ideally randomized, but *HSP* gene expression patterns (Figure S6), which were known to be heat-responsive, suggest that the effect of elevated temperature was detected in this analysis. Although the possibility that the effect of container (*e.g.*, water amount, specific density) influenced the gene expression could not be denied, we postulated that it was limited.

The differential expression patterns of NPC2-type sterol transporter genes further corroborated that our experimental settings were comparable to previous studies, which showed that a gene family member *NPC2D* was up-regulated in the symbiotic state compared to the apo-symbiotic state in *E. diaphana* (Lehnert *et al.* 2014; Baumgarten *et al.* 2015) (Figure S3). In addition, *E. diaphana NPC2B*, *C*, *D*, and *F* were closely related to *NPC2-d* in the snakelocks anemone *Anemonia viridis* (Figure S4), which was shown to be preferentially accumulated in the gastrodermal cells at the mRNA and protein levels and significantly down-regulated in response to heat stress (Dani *et al.* 2014). Most *NPC2* genes were differentially expressed, except between Apo-Norm and Apo-Heat (Figure S3, S4). This may be related to the finding that the *NPC2* gene expression was less sensitive to heat in the symbiont-free epidermis cells in *A. viridis* (Dani *et al.* 2014).

Previous studies showed that well-known stress response genes encoding HSPs were differentially expressed in response to heat stress in the corals *Orbicella* (formerly *Montastraea*) *faveolata* (DeSalvo *et al.* 2008) and *Acropora palmata* (DeSalvo *et al.* 2010), but no significant DEGs were detected in the temperate sea anemone *Anthopleura elegantissima* (Richier *et al.* 2008). In the case of *E. diaphana*, a major heat stress responsive chaperone *H90A1* (Picard 2002) was up-regulated regardless of symbiotic state, while different HSP genes were differentially expressed solely in either symbiotic state (Figure S6). Overall, these results imply the presence of multiple pathways for regulating the expression of genes, including HR-DEGs, in a symbiotic state- and/or species-dependent manner.

To further investigate the gene expression regulation and functional properties of HR-DEGs, we conducted GO term enrichment analysis. (Figure 2, Figure S5, Figure S7). The results suggest that some functions (*e.g.*, oxidation-reduction process and transmembrane transporter activity) are enriched in both the shared HR-DEGs and symbiotic-specific HR-DEGs. For instance, as previous studies showed that sea anemone and coral expel living symbionts as a pellet wrapped with the host mucus (Steele 1977) by exocytosis (Davy *et al.* 2012), heat stress may undermine the regulation of mucus rearrangement and resynthesis using genes related to extracellular matrix functioning (*e.g.*, collagen alpha *COLA1* and *CO4A2*, Fibrillin *FBN1* and *FBN2*) when the host accommodates symbionts (Figure 2, Figure S7).

### Genes and functions associated with ‘heat-induced bleaching’

**Figure 5:**
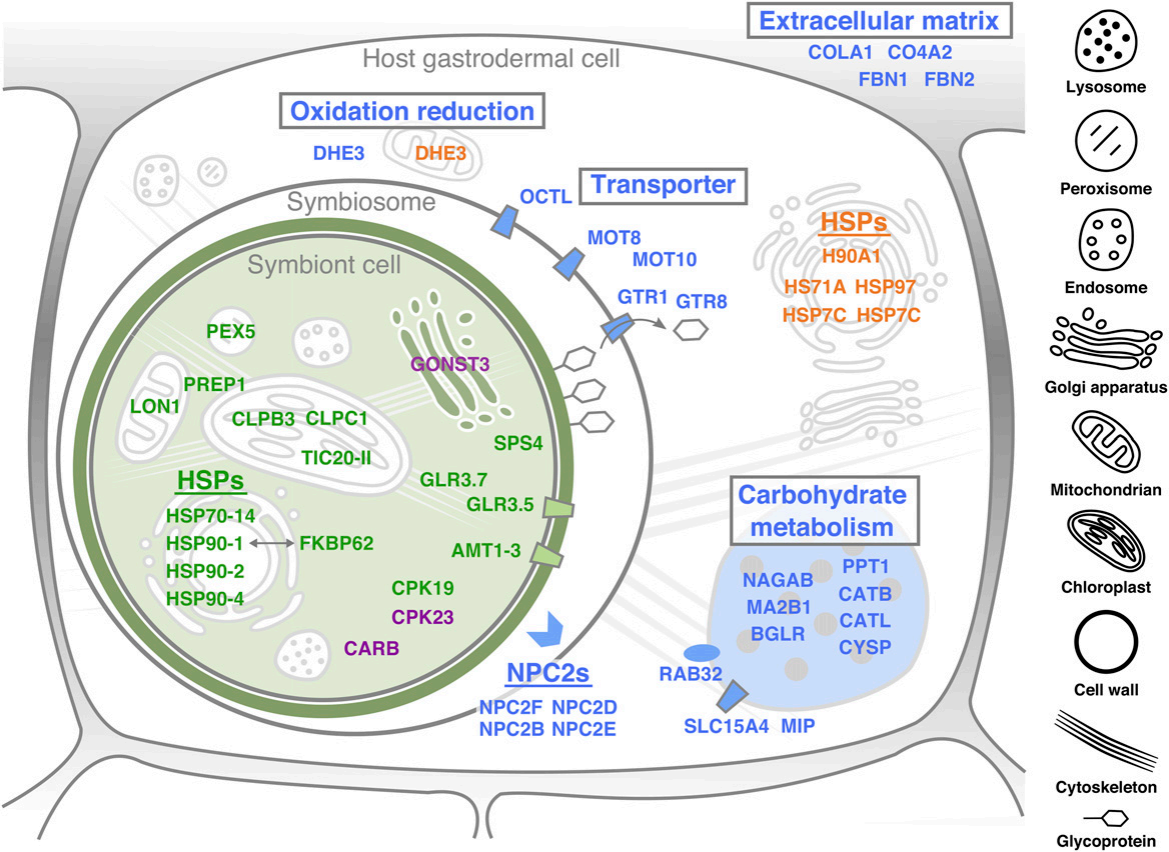
A model of the molecular interplay between host and symbiont under heat stress. Text color indicates up-regulated (orange and purple) and down-regulated (blue and green) expression in the host and symbiont cells, respectively. Field color and boxed text indicate organelles and gene functions potentially involved in heat stress response, respectively (see Discussion). Data are from Figure 3, 4, S5, S7, Table S1, S2, S4, S5.

GO term enrichment analysis using the HIBA genes identified four functional groupings (Figure 3C). Considering that the HIBA genes were important candidates for their role in bleaching, these results could provide insights into the cellular and molecular functions involved in this process. Most of the genes linked with carbohydrate metabolism (*e.g.*, Alpha-N-acetylgalactosaminidase *NAGAB*, Lysosomal alpha-mannosidase *MA2B1*) were involved in degradation and/or modification of complex carbohydrates such as *N*-linked glycosylation of glycoprotein, which are generally generated in the Golgi apparatus and transported to a lysosome (Table S4) (Winchester 2005). Meanwhile, many of the genes associated with the term ‘lysosome’ were related to lysosomal protein modification or protease activities, *e.g.*, Beta-glucuronidase *BGLR*, Palmitoyl-protein thioesterase 1 *PPT1*, Cathepsin proteases *CATB*, *CATL*, *CYSP* (Table S4). A lysosome is a versatile organelle and in normal conditions it is likely that the HIBA genes are involved in multiple functions such as regulating turnover rates of host proteins. In the HIB process, lysosomal degradation and modification functions may be suppressed by down-regulating the HIBA genes (Figure 5).

In the cnidarian-algal symbiosis, symbionts are maintained in symbiosomes within gastrodermal cells of the host (Wakefiel and Kempf 2001). Symbiodiniaceae symbionts in sea anemones and corals can be digested in certain conditions (Bieri *et al.* 2016), presumably via fusion with the lysosome- and autophagy-mediated degradation (Weis 2008). Additionally, it was reported that in the *Hydra-Chlorella* symbiosis, unhealthy symbionts might be swept out via fusion of lysosomes with symbiosomes (Hohman *et al.* 1982). In regulating lysosome-phagosome fusion, the Rab GTPase gene family, an important regulator of vesicular trafficking (Schwartz *et al.* 2007), has been proposed to play key roles. Previous studies suggested that Rab family proteins were localized in phagosomes and are possibly involved in the exclusion and maintenance of symbionts in *Aiptasia pulchella* (a synonym of *E. diaphana*) (Chen *et al.* 2005; Hong *et al.* 2009). In our data, the HIBA genes included a gene encoding Rab32, which is a regulator of the lysosomal enzyme recruitment to phagosome (Seto *et al.* 2011); the transcription regulation may be relevant to the symbiosome maintenance (Figure S8).

Another functional group in the HIBA genes is ‘transporter,’ including facilitated glucose transporters *GTR1* (*GLUT1*) and *GTR8* (*GLUT8*), monocarboxylate transporters *MOT8* and *MOT10. GTR8* is proposed to be a potential symbiosome-localized transporter that transfers glucose from the symbiont to the host (Sproles *et al.* 2018), and perhaps other HIBA transporters may also be associated with phagosome and/or symbiosome. Proton oligopeptide cotransporter SLC15A4 and aquaporin-like MIP proteins were predicted to be localized to internal membrane by MemPype, while seven other transporter candidates were not. Further biochemical studies are needed.

The other HIBA group ‘oxidation-reduction’ included two closely related *DHE3* genes encoding a cnidarian-specific subtype of glutamate dehydrogenase (Figure S9A), which are only distantly related to another homolog (AIPGENE3776) belonging to the canonical mitochondrion-targeted *DHE3* gene family widely conserved in eukaryotes. Notably, the transcription of these two genes was regulated in the opposite direction (Figure 3C), and iPSORT program predicted a mitochondrial targeting signal in the N-terminal amino acid sequence of up-regulated gene, but not in the down-regulated one (Figure S9B), suggesting differential transcriptional regulation for closely related homologs localized in different intracellular compartments (Figure 5) (Mastorodemos *et al.* 2009).

### The symbiont transcriptomic responses induced by heat stress in hospite

Our results showed that some *HSP* gene family members (*e.g.*, *HSP70-14*, *HSP90-1*, *90-2*, *90-4*) constitute major components of the symbiont HR-DEGs when *Breviolum* symbionts were hosted by *E. diaphana* (Figure 4, Table S5). A previous study demonstrated that thermal stress did not induce substantial global changes in the transcriptomes of the symbiont *Durusdinium* spp. (clade D *Symbiodinium*) colonized in the coral *Acropora hyacinthus*; whereas, a few genes encoding HSPs were weakly differentially expressed in response to heat stress (Barshis *et al.* 2014). Another study showed that *HSP70* and *HSP90* genes in *Cladocopium* (clade C) colonized in the coral *Acropora millepora* were differentially expressed in response to heat stress, depending on the acuteness of the heat treatment (Rosic *et al.* 2011). These results collectively suggest that symbiont *HSP* gene expression was differentially regulated by multiple factors, including environmental and phylogenetic constraints. *FKB62*, another symbiont HR-DEG, encoded a peptidyl prolyl isomerases FKBP62 (Figure 4, Table S5), which plays a role in high temperature tolerance by interacting with HSP90.1 and stabilizing small HSPs in *Arabidopsis* (Meiri and Breiman 2009). Our data showed that *FKB62* as well as many *HSPs* in the symbionts were down-regulated in response to heat (Figure 4), in contrast to the up-regulation of host *HSPs* (Figure S6), implying that symbiont *HSPs* might negatively regulate the heat responsive gene expression, as proposed in a previous study (Rosic *et al.* 2011).

Found in the symbiont HR-DEG were a component of the inner chloroplast membrane translocon (TIC) complex *TIC20*, chloroplastic/mitochondrial presequence protease 1 *PREP1*, chloroplastic chaperone proteins *ClpC1* and *ClpB3*, a mitochondrial Lon protease homolog *LON1*, and an import receptor for peroxisomal-targeting signal peptide *PEX5*; this result suggests that proper regulation of protein import from cytosol to organellar compartments was inhibited in symbionts under heat stress. In cytosol, the following symbiont HR-DEGs may be directly or indirectly involved in calcium signaling and affected by heat stress: glutamate-gated cation channel proteins *GLR3.5* and *GLR3.7*, calcium-dependent protein kinases *CPK19* and *CPK23*, a plasma membrane-localized ammonium transporter *AMT1-3*, a carbamoyl phosphate synthetase B *CARB* (Table S5) (Michaeli and Fromm 2015). As ammonium assimilation is a core process in the nitrogen cycling and amino acid relocation between cnidarian hosts and their symbionts (Pernice *et al.* 2012), genes involved in this process such as *AMT1-3* and *CARB* may also play a key role in molecular interactions in the nitrogen-limited oligotrophic oceans, via balancing the carbon/nitrogen ratio in the symbiont cells (Houlbrèque and Pagès 2009).

The symbiont HR-DEGs contain two sugar metabolism-related genes. One is a gene encoding a nucleotide-sugar transporter *GONST3*, which may function in the import of nucleotide-sugar from cytosol to the Golgi apparatus for downstream glycosylation reactions. The other encodes a sucrose-phosphate synthase family protein *SPS4*, which might be related to photosynthetic sucrose synthesis. In the cnidarian-algal symbiosis, it is suggested that sugar, more specifically glucose, is an important component for not only the supply of photosynthesized carbohydrates from symbiont to host (Burriesci *et al.* 2012) but also for the recognition of symbionts by the host (Takeuchi *et al.* 2017; Huang *et al.* 2017). Our results raise a possibility that cytosolic sugar metabolism and Golgi apparatus-mediated glycosylation of proteins and/or cell wall components may be susceptible to stress and damage when symbionts are exposed to heat *in hospite* (Figure 5).

### A hypothesis on the molecular interplay between host and symbiont under heat stress

Our data pinpoint that, in addition to the differences of steady state transcriptomes, cnidarian hosts possessing the same genetic background can respond to the same environmental changes, such as heat stress, in very different ways depending on their symbiotic state (Figures 1, 2). Furthermore, we identified HIBA genes associated with the symbiotic state transition and showed novel predicted functions of potential importance in symbiosome maintenance (Figure 5).

One plausible hypothesis is that the HIBA genes play key roles in lysosomal (or symbiosomal) degradation and modification of glycoproteins at the symbiont cell surface (Winchester 2005) and thereby affecting the symbiosis stability under heat stress (Figure 5). Previous studies suggested that lectin proteins capable of binding the glucose moiety might be involved in the recognition of Symbiodiniaceae symbionts by the host coral *Acropora tenuis* (Takeuchi *et al.* 2017). Furthermore, a glycoprotein was characterized as the first Symbiodiniaceae protein and was localized at the cell surface, expressed exclusively when the symbiont was colonized within the host (Huang *et al.* 2017). In the HIB process, the altered transport rate of degraded metabolites to the host cytosol may work as a negative feedback signal for the subsequent decrease of metabolite flow (Davy *et al.* 2012). Further investigation of the molecular interaction between host and symbiont, presumably mediated via glycoprotein metabolism in lysosomes and symbiosomes, will be key to understanding what signal can trigger the collapse of symbiosis.
